# Supplementary material for: A quantitative map of nuclear pore assembly reveals two distinct mechanisms
Source: Nature. 2023 Jan 4;613(7944):575–81. doi: 10.1038/s41586-022-05528-w (PMC9849139; doi:10.1038/s41586-022-05528-w)

nature portfolio

https://doi.org/10.1038/s41586-022-05528-w

Supplementary information

A quantitative map of nuclear pore assembly reveals two distinct mechanisms

In the format provided by the
authors and unedited

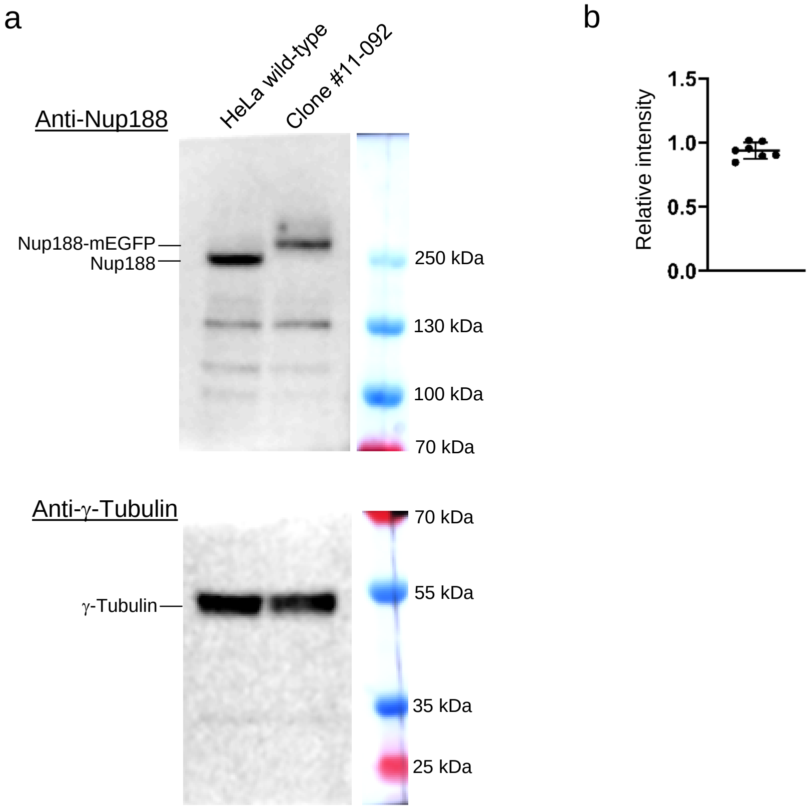

Uncropped immunoblot data for Nup188-mEGFP genome-edited cells. (a) The membrane was cut at the position of 70 kDa, and the membrane above 70 kDa was blotted with an antibody against Nup188 (top) and the membrane below 70 kDa was blotted with an antibody against γ-Tubulin (bottom). The clone 11-092 is the cell line that is used in this study. (b) The relative intensity of the bands for GFP-tagged Nup188 over endogenous Nup188. The plot is from 7 pairs of bands (GFP-tagged vs endogenous) from 3 independent experiments. The mean is depicted as a horizontal line and the whiskers show the standard deviation.

Supplementary Fig. 1

Supplementary Fig. 2: The reaction scheme used for the mathematical modelling for the nuclear pore assembly kinetics.

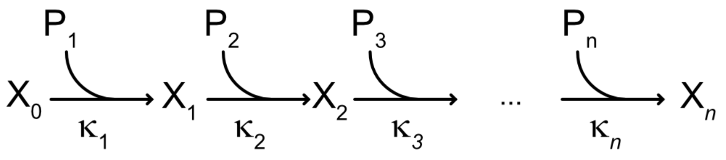

Supplement: Supplementary file 1 — Supplementary Figures [file 41586_2022_5528_MOESM1_ESM.pdf]
