# Supplementary material for: A quantitative map of nuclear pore assembly reveals two distinct mechanisms
Source: Nature. 2023 Jan 4;613(7944):575–81. doi: 10.1038/s41586-022-05528-w (PMC9849139; doi:10.1038/s41586-022-05528-w)
Supplement: Supplementary file 2 — Reporting Summary [file 41586_2022_5528_MOESM2_ESM.pdf]

nature portfolio

Corresponding author(s): Shotaro Otsuka, Jan Ellenberg

Last updated by author(s): Oct 22, 2022

Reporting Summary

Nature Portfolio wishes to improve the reproducibility of the work that we publish. This form provides structure for consistency and transparency in reporting. For further information on Nature Portfolio policies, see our Editorial Policies and the Editorial Policy Checklist.

Statistics

For all statistical analyses, confirm that the following items are present in the figure legend, table legend, main text, or Methods section.

n/a	Confirmed	
☐	☒	The exact sample size (*n*) for each experimental group/condition, given as a discrete number and unit of measurement
☐	☒	A statement on whether measurements were taken from distinct samples or whether the same sample was measured repeatedly
☐	☒	The statistical test(s) used AND whether they are one- or two-sided<br>*Only common tests should be described solely by name; describe more complex techniques in the Methods section.*
☒	☐	A description of all covariates tested
☐	☒	A description of any assumptions or corrections, such as tests of normality and adjustment for multiple comparisons
☐	☒	A full description of the statistical parameters including central tendency (e.g. means) or other basic estimates (e.g. regression coefficient) AND variation (e.g. standard deviation) or associated estimates of uncertainty (e.g. confidence intervals)
☐	☒	For null hypothesis testing, the test statistic (e.g. *F*, *t*, *r*) with confidence intervals, effect sizes, degrees of freedom and *P* value noted<br>*Give P values as exact values whenever suitable.*
☐	☒	For Bayesian analysis, information on the choice of priors and Markov chain Monte Carlo settings
☒	☐	For hierarchical and complex designs, identification of the appropriate level for tests and full reporting of outcomes
☐	☒	Estimates of effect sizes (e.g. Cohen's *d*, Pearson's *r*), indicating how they were calculated

Our web collection on statistics for biologists contains articles on many of the points above.

Software and code

Policy information about availability of computer code

Data collection	ZEN software 2012 SP1 (8,1,0,484)
Data analysis	ImageJ 1.53q, Amira 5.4.5, Fluctuation Analyzer 4G 150223 (https://www-ellenberg.embl.de/resources/data-analysis), FCSFitM v0.8 (https://git.embl.de/grp-ellenberg/FCSAnalyze), FCSCalibration v0.4.2 (https://git.embl.de/grp-ellenberg/FCSAnalyze), RStudio 1.1.383, R 3.4.1, MATLAB 2017a, Python v3.6.8, Integrative Modeling Platform (IMP) v.2.13.0, gmconvert v3.0, custom code in Github repository (https://github.com/integrativemodeling/npcassembly), ChimeraX v1.1.1.

For manuscripts utilizing custom algorithms or software that are central to the research but not yet described in published literature, software must be made available to editors and reviewers. We strongly encourage code deposition in a community repository (e.g. GitHub). See the Nature Portfolio guidelines for submitting code & software for further information.

Data

Policy information about availability of data

All manuscripts must include a data availability statement. This statement should provide the following information, where applicable:

- Accession codes, unique identifiers, or web links for publicly available datasets
- A description of any restrictions on data availability
- For clinical datasets or third party data, please ensure that the statement adheres to our policy

Fluorescence images were deposited to the Image Data Resource (IDR; https://idr.openmicroscopy.org/) under accession number idr0115. Our integrative

spatiotemporal model of the postmitotic assembly of the human NPC is deposited to the nascent database of integrative structures PDB-Dev (https://pdb-dev.wwpdb.org/) under accession number PDBDEV_00000142. All source code is accessible on Github repository (for quantifying Nup intensity in core and non-core regions: https://github.com/mjh1m22/Quantitative_map_nups_Otsuka_Nature_2022; for decomposing postmitotic and interphase assembly kinetics; https://github.com/manerotoni/npc_assembly_Otsuka_2022). Integrative Modeling Platform (IMP) is an open source program freely available under the LGPL license at http://integrativemodeling.org; all input files, scripts, and output files are available at http://integrativemodeling.org/npcassembly.

Human research participants

Policy information about studies involving human research participants and Sex and Gender in Research.

Reporting on sex and gender	*Use the terms sex (biological attribute) and gender (shaped by social and cultural circumstances) carefully in order to avoid confusing both terms. Indicate if findings apply to only one sex or gender; describe whether sex and gender were considered in study design whether sex and/or gender was determined based on self-reporting or assigned and methods used. Provide in the source data disaggregated sex and gender data where this information has been collected, and consent has been obtained for sharing of individual-level data; provide overall numbers in this Reporting Summary. Please state if this information has not been collected. Report sex- and gender-based analyses where performed, justify reasons for lack of sex- and gender-based analysis.*
Population characteristics	*Describe the covariate-relevant population characteristics of the human research participants (e.g. age, genotypic information, past and current diagnosis and treatment categories). If you filled out the behavioural & social sciences study design questions and have nothing to add here, write "See above."*
Recruitment	*Describe how participants were recruited. Outline any potential self-selection bias or other biases that may be present and how these are likely to impact results.*
Ethics oversight	*Identify the organization(s) that approved the study protocol.*

Note that full information on the approval of the study protocol must also be provided in the manuscript.

Field-specific reporting

Please select the one below that is the best fit for your research. If you are not sure, read the appropriate sections before making your selection.

☒ Life sciences ☐ Behavioural & social sciences ☐ Ecological, evolutionary & environmental sciences

For a reference copy of the document with all sections, see nature.com/documents/nr-reporting-summary-flat.pdf

Life sciences study design

All studies must disclose on these points even when the disclosure is negative.

Sample size	Sample sizes were based on pilot experiments to determine the number of cells required to observe stable population averages with high Pearson's correlation between replicates. Sample sizes are indicated in figure legends.
Data exclusions	Videos of dividing cells with rotating nuclei are removed from the analysis, because we cannot properly assign the non-core and core regions.
Replication	For quantitative imaging in Fig. 1a, d, the data were from 4, 4, 4, 2, 3, 2, 3, and 2 independent experiments for Nup107, Seh1, Nup205, Nup93, Nup62, Nup214, Tpr and Nup358, respectively. STED imaging in Fig. 4a and Extended Fig. 4a and live imaging in Extended Fig. 10 were from 2 independent experiments. For dynamic quantitative imaging in Fig. 3, the data were from 4, 4, 4, 2, 3, 4, 3, 2, 2, and 4 independent experiments for Nup107, Seh1, Nup205, Nup93, Nup62, Nup214, Tpr, Nup358, Nup153, and Pom121, respectively. STED imaging in Fig. 1b, c and Fig. 4b, southern blotting in Extended Data Fig. 1 and immuno-fluorescence microscopy in Extended Data Fig. 9 are from single experiments.
Randomization	No randomization was done, because this study does not involve animals or human participants. Samples were organized into groups based on cell lines. Cells were imaged in randomly chosen fields of view per experiment. All imaged cells were further analyzed. Appropriate controls were included in all experiments.
Blinding	No predefined group allocation was performed. All data that passed QC (described in data exclusion section above) were analyzed. The analysis was performed in a reproducible and automated fashion for all experiments as described in Methods.

Reporting for specific materials, systems and methods

We require information from authors about some types of materials, experimental systems and methods used in many studies. Here, indicate whether each material, system or method listed is relevant to your study. If you are not sure if a list item applies to your research, read the appropriate section before selecting a response.

Materials & experimental systems

n/a	Involved in the study
☐	☒ Antibodies
☐	☒ Eukaryotic cell lines
☒	☐ Palaeontology and archaeology
☒	☐ Animals and other organisms
☒	☐ Clinical data
☒	☐ Dual use research of concern

Methods

n/a	Involved in the study
☒	☐ ChIP-seq
☒	☐ Flow cytometry
☒	☐ MRI-based neuroimaging

Antibodies

Antibodies used

Mouse anti-Nup62 (Cat. No. 610497; BD Biosciences, Franklin Lakes, NJ), anti-Tpr (Cat. No. HPA019661; The Human Protein Atlas), a GFP-nanobody (FluoTag®-X4 anti-GFP nanobody Abberior® Star 635P; Cat. No. N0304-Ab635P-L; NanoTag Biotechnologies, Göttingen, Germany), rabbit anti-Nup155 (Cat. No. HPA037775; The Human Protein Atlas), rabbit anti-Elys (Cat. No. HPA031658; The Human Protein Atlas), Abberior® STAR RED-conjugated goat anti-mouse IgG (Cat. No. 2-0002-011-2, Abberior GmbH, Göttingen, Germany), Abberior STAR635P goat anti-rabbit IgG (Cat. No. ST635P-1002-500UG, Abberior GmbH), and Alexa Fluor 594 goat anti-rabbit IgG (Cat. No. A-11037; Life Technologies), rabbit anti-Nup188 (Catalog No. A302-322A, Bethyl Laboratories, Montgomery, TX), rabbit anti-Nup205 (Catalog No. ab157090, Abcam, Cambridge, UK), anti-Nup358 (Catalog No. HPA023960, The Human Protein Atlas), rabbit anti-gamma-Tubulin (Cat. No. T5192, Sigma Aldrich), mouse anti-gamma-Tubulin (Cat. No. T5326, Sigma Aldrich), anti-Vinculin (Cat. No. ab219649, Abcam), anti-rabbit IgG horseradish peroxidase (HRP)-conjugated secondary (Cat. No. W4011, Promega), anti-mouse HPR-conjugated secondary (Catalog No. 040-655, Bio-Techne) and anti-rabbit HPR-conjugated secondary (Catalog No. 040-656, Bio-Techne) antibodies.

Validation

Antibodies for genome editing are validated by Western Blot for sensitivity and specificity (see Koch et al. Nature Protocols, vol. 13: 1465–1487 (2018), doi: 10.1038/nprot.2018.042).

Eukaryotic cell lines

Policy information about cell lines and Sex and Gender in Research

Cell line source(s)

Wildtype HeLa Kyoto cells (RRID: CVCL_1922) were a kind gift from Prof. Narumiya, Kyoto University. All the cell lines used in this study are derivatives of this parental HeLa cell line.

Authentication

HeLa Kyoto cells were authenticated by sequencing.

Mycoplasma contamination

Cells were screened for mycoplasma contamination by PCR before use. It was always negative.

Commonly misidentified lines
(See ICLAC register)

Cell line is not listed in the list of commonly misidentified cell lines.
