# Supplementary material for: A quantitative map of nuclear pore assembly reveals two distinct mechanisms
Source: Nature. 2023 Jan 4;613(7944):575–81. doi: 10.1038/s41586-022-05528-w (PMC9849139; doi:10.1038/s41586-022-05528-w)
Supplement: Supplementary file 4 — Concentration of nucleoporins (nM). Concentration of mEGFP-tagged nucleoporins in interphase and metaphase. Data represent mean ± S.D. from the number of cells indicated in parentheses. [file 41586_2022_5528_MOESM4_ESM.docx]

|  |  | Interphase |  | Metaphase | |
| --- | --- | --- | --- | --- | --- |
|  | Cytoplasm | Nucleoplasm | Nuclear envelope | Cytosol | Nucleoplasm |
| Nup107 | 62 ± 17 (133) | 93 ± 20 (133) | 650 ± 63 (47) | 200 ± 33 (49) | 240 ± 36 (12) |
| Seh1 | 61 ± 16 (31) | 56 ± 14 (32) | 560 ± 61 (37) | 180 ± 26 (20) | 250 ± 28 (12) |
| Nup205 | 15 ± 7.9 (34) | 23 ± 7.7 (34) | 350 ± 54 (41) | 90 ± 14 (21) | 59 ± 5.1 (10) |
| Nup93 | 60 ± 21 (12) | 65 ± 12 (12) | 860 ± 110 (20) | 320 ± 29 (10) | 210 ± 11 (8) |
| Nup62 | 170 ± 52 (28) | 84 ± 27 (28) | 1000 ± 210 (41) | 400 ± 54 (15) | 240 ± 22 (11) |
| Nup214 | 34 ± 12 (16) | 22 ± 2.8 (16) | 450 ± 70 (26) | 120 ± 26 (15) | 68 ± 7.9 (18) |
| Tpr | 6.2 ± 8.3 (12) | 45 ± 18 (12) | 420 ± 63 (55) | 120 ± 20 (10) | 84 ± 19 (9) |
| Nup358 | 13 ± 7.5 (6) | 9.7 ± 3.4 (6) | 390 ± 61 (28) | 88 ± 18 (10) | 65 ± 5.0 (14) |
