# Supplementary material for: A quantitative map of nuclear pore assembly reveals two distinct mechanisms
Source: Nature. 2023 Jan 4;613(7944):575–81. doi: 10.1038/s41586-022-05528-w (PMC9849139; doi:10.1038/s41586-022-05528-w)
Supplement: Supplementary file 5 — gRNA sequences used for genome editing. The first sequence is the antisense gRNA-binding site, the second one is the sense gRNA-binding site. For Nup188, Alt-R S.p. HiFi Cas9 Nuclease V3 was used that requires only single gRNA. [file 41586_2022_5528_MOESM5_ESM.docx]

| Gene | Sequences |
| --- | --- |
| Nup153 | AGGAGGCCGAGGGCTCCGGT  CGGACGCGGCGTTGCCACCA |
| Nup93 | TGTCAGTACATCAGGCACAT  AGCATGGCACTTAATTCATG |
| Nup188 | TGTCCGGCATATGCAAAGATAGG |
|  |  |
| Pom121 | GGACAAAGGCTACTTTTTGC  CCTTCCCTAAATCTGGACCT |
| Seh1 | CCACAAACATGGTTTCCGTC  CAGCATCGCGGCGGACCACA |
