# Supplementary material for: A quantitative map of nuclear pore assembly reveals two distinct mechanisms
Source: Nature. 2023 Jan 4;613(7944):575–81. doi: 10.1038/s41586-022-05528-w (PMC9849139; doi:10.1038/s41586-022-05528-w)
Supplement: Supplementary file 6 — Parameter of the mathematical model. The number in brackets represents the 95% confidence interval as estimated using profile likelihood. *The fraction of postmitotic assembly in the non-core and core region represent the average for all Nups. See Methods. The assembly in the core region is delayed by dc = 2 min. [file 41586_2022_5528_MOESM6_ESM.docx]

| **Nup** | **Postmitotic assembly** | | | **Interphase assembly** | | | **Fraction post-mitotic*** | |
| --- | --- | --- | --- | --- | --- | --- | --- | --- |
|  | ***n_p_*** | ***K_p_* (min)** | **Δ*T_p_* (min)** | ***n_i_*** | ***K_i_* (min)** | **Δ*T_i_* (min)** | ***f_n_*_,_ non-core** | ***f*_c_, core** |
| Nup107 | 3.38 [2.67, 4.42] | 6.51 [5.95, 7.13] | 9.1 [6.69, 11.99] | 3.48 [2.72, 4.56] | 36.17 [32.87, 39.7] | 48.72 [36.73, 65.61] | 0.857 [0.76, 0.95] | 0.295 [0.17, 0.4] |
| Seh1 | 3.3 [2.67, 4.21] | 7.25 [6.61, 7.98] | 10.39 [7.77, 13.54] | 2.78 [2.16, 3.58] | 37.36 [33.45, 41.83] | 65.41 [48.96, 92.83] |  |  |
| Nup205 | 6.73 [5.11, 9.4] | 7.91 [7.41, 8.51] | 5.25 [3.62, 7.3] | 4.3 [3.33, 5.78] | 47.73 [43.8, 52.16] | 50.93 [37.11, 69.41] |  |  |
| Nup93 | 5.71 [4.84, 6.96] | 8.84 [8.4, 9.31] | 6.97 [5.59, 8.46] | 3.94 [3.19, 4.97] | 48.66 [45.42, 52.37] | 57.16 [44.15, 74.26] |  |  |
| Nup62 | 4.71 [4.03, 5.6] | 9.6 [9.11, 10.14] | 9.29 [7.56, 11.27] | 5.53 [4.31, 7.51] | 45.8 [42.85, 49.39] | 37.34 [26.53, 50.54] |  |  |
| Nup214 | 3.13 [2.79, 3.6] | 14.83 [13.61, 16.08] | 22.54 [18.24, 26.91] | 2.61 [2.05, 3.66] | 41.12 [36.32, 47.71] | 77.68 [52.38, 115.29] |  |  |
| Tpr | 5.35 [4.64, 6.29] | 16.37 [15.66, 17.15] | 13.83 [11.58, 16.3] | 2.56 [2.05, 3.22] | 34.88 [30.66, 41.46] | 67.46 [48.48, 102.15] |  |  |
| Nup358 | 2.54 [2.17, 3] | 24.55 [22.6, 26.78] | 48.02 [38.54, 60.61] | 4.88 [3.63, 6.7] | 64.13 [59.28, 70.9] | 59.76 [42.21, 87.08] |  |  |
| Nup153 | 5.67 [3.97, 9.14] | 6.15 [5.64, 6.74] | 4.89 [2.97, 7.23] | 1.83 [1.45, 2.32] | 35.27 [30.47, 44.88] | 106.24 [71.42, 183.97] |  |  |
| Pom121 | 6.38 [3.89, 17.09] | 6.39 [5.48, 7.46] | 4.49 [1.63, 7.65] | 2.41 [1.64, 3.65] | 28.25 [23.19, 36.07] | 58.98 [35.46, 114.84] |  |  |
|  | | | | | | | | |
